# Supplementary material for: HIVprotI: an integrated web based platform for prediction and design of HIV proteins inhibitors
Source: J Cheminform. 2018 Mar 9;10:12. doi: 10.1186/s13321-018-0266-y (PMC5845081; doi:10.1186/s13321-018-0266-y)
Supplement: Supplementary file 2 — Additional file 2. Source code of HIVProtI web server. [file 13321_2018_266_MOESM2_ESM.zip › HIVprotI_Source-code/hivprotI/weka-3-6-6/doc/weka/experiment/CSVResultListener.html]

CSVResultListener


---


|  |  |  |
| --- | --- | --- |
| **Overview** \| **Package** \| **Class** \| **Tree** \| **Deprecated** \| **Index** \| **Help** \| **Weka's home** | |  |
| **PREV CLASS**   **NEXT CLASS** | **FRAMES**    **NO FRAMES**     **All Classes** |  |
| SUMMARY: NESTED \| FIELD \| CONSTR \| METHOD | DETAIL: FIELD \| CONSTR \| METHOD |  |


---


## weka.experiment Class CSVResultListener

```
java.lang.Object
  weka.experiment.CSVResultListener
```

**All Implemented Interfaces:**: java.io.Serializable, OptionHandler, RevisionHandler, ResultListener

**Direct Known Subclasses:**: InstancesResultListener

---

``` public class CSVResultListener extends java.lang.Object implements ResultListener, OptionHandler, RevisionHandler ```

Takes results from a result producer and assembles them into comma separated value form.


Valid options are:

```
 -O <file name>
  The filename where output will be stored. Use - for stdout.
  (default temp file)
```

**Version:**
:   $Revision: 1.13 $

**Author:**
:   Len Trigg (trigg@cs.waikato.ac.nz)

**See Also:**: Serialized Form

---

**Constructor Summary**

`CSVResultListener()`
  
          Sets temporary file.


| **Method Summary** | |
| --- | --- |
| `void` | `acceptResult(ResultProducer rp, java.lang.Object[] key, java.lang.Object[] result)`             Just prints out each result as it is received. |
| `java.lang.String[]` | `determineColumnConstraints(ResultProducer rp)`             Determines if there are any constraints (imposed by the destination) on the result columns to be produced by resultProducers. |
| `java.lang.String[]` | `getOptions()`             Gets the current settings of the Classifier. |
| `java.io.File` | `getOutputFile()`             Get the value of OutputFile. |
| `java.lang.String` | `getRevision()`             Returns the revision string. |
| `java.lang.String` | `globalInfo()`             Returns a string describing this result listener |
| `boolean` | `isResultRequired(ResultProducer rp, java.lang.Object[] key)`             Always says a result is required. |
| `java.util.Enumeration` | `listOptions()`             Returns an enumeration describing the available options.. |
| `java.lang.String` | `outputFileName()`             Get the value of OutputFileName. |
| `java.lang.String` | `outputFileTipText()`             Returns the tip text for this property |
| `void` | `postProcess(ResultProducer rp)`             Perform any postprocessing. |
| `void` | `preProcess(ResultProducer rp)`             Prepare for the results to be received. |
| `void` | `setOptions(java.lang.String[] options)`             Parses a given list of options. |
| `void` | `setOutputFile(java.io.File newOutputFile)`             Set the value of OutputFile. |
| `void` | `setOutputFileName(java.lang.String name)`             Set the value of OutputFileName. |

**Methods inherited from class java.lang.Object**

`equals, getClass, hashCode, notify, notifyAll, toString, wait, wait, wait`

**Constructor Detail**

### CSVResultListener

```
public CSVResultListener()
```

:   Sets temporary file.


**Method Detail**

### globalInfo

```
public java.lang.String globalInfo()
```

:   Returns a string describing this result listener

    :   **Returns:**: a description of the result listener suitable for displaying in the explorer/experimenter gui

---


### listOptions

```
public java.util.Enumeration listOptions()
```

:   Returns an enumeration describing the available options..

    :   **Specified by:**: `listOptions` in interface `OptionHandler`
    :   **Returns:**: an enumeration of all the available options.

---


### setOptions

```
public void setOptions(java.lang.String[] options)
                throws java.lang.Exception
```

:   Parses a given list of options.


    Valid options are:

    ```
     -O <file name>
      The filename where output will be stored. Use - for stdout.
      (default temp file)
    ```

    :   **Specified by:**: `setOptions` in interface `OptionHandler`
    :   **Parameters:**: `options` - the list of options as an array of strings **Throws:**: `java.lang.Exception` - if an option is not supported

---


### getOptions

```
public java.lang.String[] getOptions()
```

:   Gets the current settings of the Classifier.

    :   **Specified by:**: `getOptions` in interface `OptionHandler`
    :   **Returns:**: an array of strings suitable for passing to setOptions

---


### outputFileTipText

```
public java.lang.String outputFileTipText()
```

:   Returns the tip text for this property

    :   **Returns:**: tip text for this property suitable for displaying in the explorer/experimenter gui

---


### getOutputFile

```
public java.io.File getOutputFile()
```

:   Get the value of OutputFile.

    :   **Returns:**: Value of OutputFile.

---


### setOutputFile

```
public void setOutputFile(java.io.File newOutputFile)
```

:   Set the value of OutputFile. Also sets the
    OutputFileName.

    :   **Parameters:**: `newOutputFile` - Value to assign to OutputFile.

---


### outputFileName

```
public java.lang.String outputFileName()
```

:   Get the value of OutputFileName.

    :   **Returns:**: Value of OutputFile.

---


### setOutputFileName

```
public void setOutputFileName(java.lang.String name)
```

:   Set the value of OutputFileName. Must be used
    AFTER setOutputFile.

    :   **Parameters:**: `name` - the name of OutputFile.

---


### preProcess

```
public void preProcess(ResultProducer rp)
                throws java.lang.Exception
```

:   Prepare for the results to be received.

    :   **Specified by:**: `preProcess` in interface `ResultListener`
    :   **Parameters:**: `rp` - the ResultProducer that will generate the results **Throws:**: `java.lang.Exception` - if an error occurs during preprocessing.

---


### postProcess

```
public void postProcess(ResultProducer rp)
                 throws java.lang.Exception
```

:   Perform any postprocessing. When this method is called, it indicates
    that no more results will be sent that need to be grouped together
    in any way.

    :   **Specified by:**: `postProcess` in interface `ResultListener`
    :   **Parameters:**: `rp` - the ResultProducer that generated the results **Throws:**: `java.lang.Exception` - if an error occurs

---


### determineColumnConstraints

```
public java.lang.String[] determineColumnConstraints(ResultProducer rp)
                                              throws java.lang.Exception
```

:   Determines if there are any constraints (imposed by the
    destination) on the result columns to be produced by
    resultProducers. Null should be returned if there are NO
    constraints, otherwise a list of column names should be
    returned as an array of Strings.

    :   **Specified by:**: `determineColumnConstraints` in interface `ResultListener`
    :   **Parameters:**: `rp` - the ResultProducer to which the constraints will apply **Returns:**: an array of column names to which resutltProducer's results will be restricted. **Throws:**: `java.lang.Exception` - if an error occurs.

---


### acceptResult

```
public void acceptResult(ResultProducer rp,
                         java.lang.Object[] key,
                         java.lang.Object[] result)
                  throws java.lang.Exception
```

:   Just prints out each result as it is received.

    :   **Specified by:**: `acceptResult` in interface `ResultListener`
    :   **Parameters:**: `rp` - the ResultProducer that generated the result: `key` - The key for the results.: `result` - The actual results. **Throws:**: `java.lang.Exception` - if the result could not be accepted.

---


### isResultRequired

```
public boolean isResultRequired(ResultProducer rp,
                                java.lang.Object[] key)
                         throws java.lang.Exception
```

:   Always says a result is required. If this is the first call,
    prints out the header for the csv output.

    :   **Specified by:**: `isResultRequired` in interface `ResultListener`
    :   **Parameters:**: `rp` - the ResultProducer wanting to generate the result: `key` - The key for which a result may be needed. **Returns:**: true if the result should be calculated. **Throws:**: `java.lang.Exception` - if it could not be determined if the result is needed.

---


### getRevision

```
public java.lang.String getRevision()
```

:   Returns the revision string.

    :   **Specified by:**: `getRevision` in interface `RevisionHandler`
    :   **Returns:**: the revision


---


|  |  |  |
| --- | --- | --- |
| **Overview** \| **Package** \| **Class** \| **Tree** \| **Deprecated** \| **Index** \| **Help** \| **Weka's home** | |  |
| **PREV CLASS**   **NEXT CLASS** | **FRAMES**    **NO FRAMES**     **All Classes** |  |
| SUMMARY: NESTED \| FIELD \| CONSTR \| METHOD | DETAIL: FIELD \| CONSTR \| METHOD |  |


---
